# A low energy paediatric clavicle fracture associated with acute brachial plexus injury and subclavian artery compression

**DOI:** 10.1308/003588413X13511609955256

**Published:** 2013-03

**Authors:** I Gill, J Quayle, M Fox

**Affiliations:** Frimley Park Hospital NHS Foundation Trust,UK

**Keywords:** Clavicle, Fracture, Brachial plexus, Low energy

## Abstract

Paediatric clavicle fractures are common injuries presenting to orthopaedic surgeons. The majority of these represent midshaft low energy fractures, which in the vast majority of cases are treated non-operatively and recover rapidly. The main indications to consider operative intervention include high energy of injury, >2cm shortening, open fractures and associated vascular or neurological injuries. Brachial plexus (BP) injuries are uncommon with variable outcomes. They often result from high energy motorcycle related accidents with potentially fatal associated injuries such as vascular disruption. Their management is complex, requiring expertise, and they are therefore usually managed in supraregional centres.

We present a unique case of a low energy midshaft clavicle fracture in a paediatric patient in whom there was an acute BP injury and subclavian artery compression that has not been described previously.

Paediatric clavicle fractures are common injuries presenting to orthopaedic surgeons. The majority of these represent midshaft low energy fractures, which in the vast majority of cases are treated non-operatively and recover rapidly. The main indications to consider operative intervention include high energy of injury, >2cm shortening, open fractures and associated vascular or neurological injuries.^1^ Brachial plexus (BP) injuries are uncommon with variable outcomes. They often result from high energy motorcycle related accidents with potentially fatal associated injuries such as vascular disruption. Their management is complex, requiring expertise, and they are therefore usually managed in supraregional centres.

We present a unique case of a low energy midshaft clavicle fracture in a paediatric patient in whom there was an acute BP injury and subclavian artery compression that has not been described previously.

## Case history

**Figure 1:**
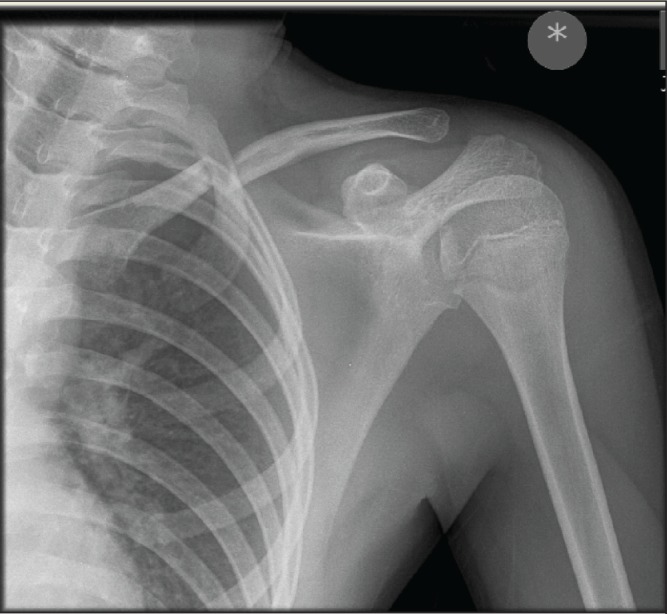
Plain x-ray showing midshaft clavicle fracture

A 13-year-old boy with no relevant past medical history was playing football with a friend. He tripped over and fell to the floor with the second boy landing on him. He had immediate pain around his left shoulder girdle and presented to the accident and emergency (A&E) department. At initial presentation, the left upper limb had no overt neurovascular deficit but there was an obvious closed deformity of the left clavicle. Plain radiography performed shortly after admission identified a midshaft clavicle fracture (Fig 1). Prior to planned discharge from the A&E department, radial nerve dysfunction (sensory and motor) was noted, prompting orthopaedic referral.

On orthopaedic review, the limb was well perfused with good radial and ulnar pulses, and normal capillary refill. Neurological assessment identified reduced sensation in the C5 and T1 dermatomes, and absent sensation in C6, C7 and C8. Motor examination suggested intact rhomboids, supraspinatus, infraspinatus and serratus anterior. Motor function in the deltoid, biceps, triceps, wrist flexors, wrist extensors, finger flexors and intrinsic muscles was absent.

Following this examination, a cervical spine injury or BP injury was suspected, and the patient was admitted for observation and further investigation. Plain radiography of the cervical spine failed to demonstrate any abnormality. Urgent magnetic resonance imaging (MRI) of the cervical spine and BP as well as computed tomography (CT) were organised.

The MRI demonstrated oedema around the clavicle fracture with no obvious injury of the cervical roots or spine. Soft vascular signs, reduced volume radial pulse, sluggish capillary refill and temperature difference were noted but no pallor or pain. Due to the presence of soft vascular signs, CT angiography was performed in preference to magnetic resonance angiography as it is superior in demonstrating fracture configuration.

**Figure 2:**
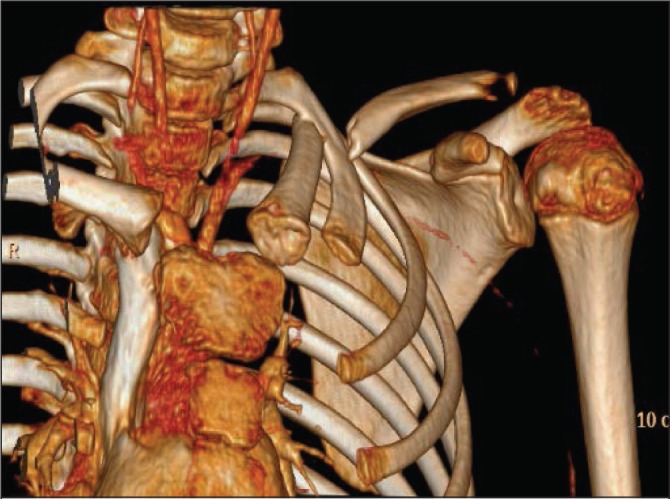
Three-dimensional computed tomography reconstruction showing posteriorly directed medial clavicle beneath first rib

**Figure 3:**
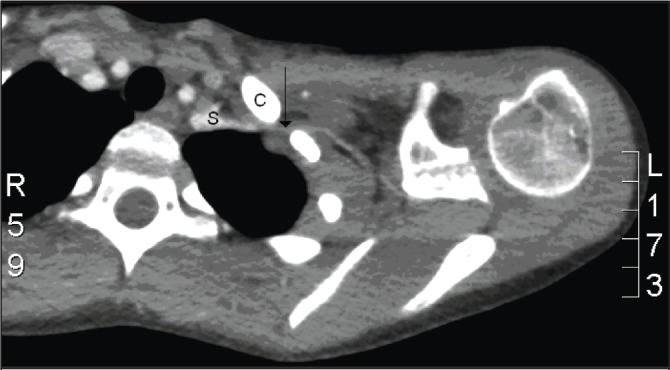
Computed tomography angiography showing the medial clavicle (C) compressing the subclavian artery (S) with significantly reduced volume (arrow)

A three-dimensional reconstruction demonstrated that the medial portion of the clavicle was directed in an anteroposterior direction with a slight superior orientation (Fig 2). The CT angiography showed that the end of the medial clavicle was compressing the subclavian artery, reducing the volume distal to this; however, there was no contrast leaking around vessel, suggesting the vessel remained intact (Fig 3).

Due to the combined BP and vascular concerns, the patient was referred to a supraregional peripheral nerve injury (PNI) centre and local vascular surgeons. However, because of difficulties in obtaining vascular cover at the supraregional centre, the PNI surgeon travelled to a referring hospital, where a combined procedure with PNI, orthopaedic and vascular surgeons occurred.

The patient underwent exploration under general anaesthesia in the beach chair position, neck extended with the upper arm to the midline and the inferior border of the mandible exposed. At this time, approximately 12 hours following presentation, the limb remained slightly cooler with reduced radial pulse, sluggish capillary refill and evidence of venous engorgement. A base of neck supraclavicular incision was combined with an infraclavicular deltopectoral approach to allow exposure of the whole BP (Fiolle and Delmas).^2^ The supraclavicular approach was technically difficult in view of the posterior displacement of the sternocleidomastoid muscle. The boundaries of the posterior triangle of the neck were closed due to the displacement of the medial clavicle fragment posteriorly.

**Figure 4:**
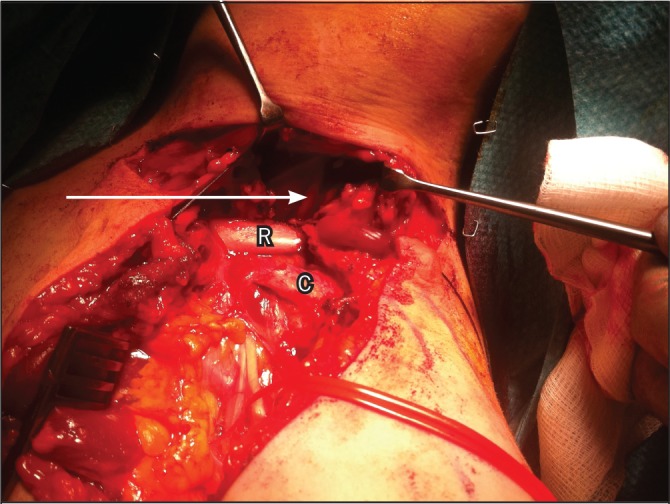
Intraoperative photograph demonstrating the medial portion of clavicle (C) directed posteriorly beneath the first rib (R) compressing trunks of the brachial plexus (arrow)

After exposing the supra and infraclavicular plexus, the lateral fragment of the clavicle was readily identified and mobilised. The medial clavicle was then carefully identified at its medial end and traced posteriorly with subperiosteal dissection. The neurovascular structures were traced either side of the clavicle (Fig 4). The entire BP and the subclavian artery were found to be compressed tightly between the first rib and the medial clavicle fragment. The subclavius muscle and its fascia were identified and felt to be in continuity running beneath the fractured medial clavicle. The medial clavicle was disimpacted from under the first rib using reduction forceps and countertraction applied through a bone hook stabilising the first rib. This manoeuvre required considerable force.

The volume of the axillary artery improved immediately and the radial pulse improved shortly thereafter. Further inspection confirmed that the trunks and divisions of the plexus remained intact deep to the subclavius muscle, albeit significantly contused. Pleural patency was confirmed with water testing. The clavicle was reduced and held with a precontoured locking plate.

The patient was discharged two days later after an uneventlful recovery. At this time, no motor function had returned but sensation had improved in the C5 and T1 dermatomes.

At the six-month follow-up appointment, the patient had made a good recovery with return of full shoulder girdle function. In addition, he had regained biceps, triceps, long flexors and extensors including flexor pollicis longus to level 3 or 4 on the Medical Research Council scale. Tinel’s sign was demonstrated at 3cm from the proximal wrist crease for the radial, ulnar and median nerves. He had no significant neuropathic pain.

## Discussion

The vast majority of clavicle fractures in children are low energy injuries that go rapidly on to uneventful union and return to function.^3^ This case represents an exceptionally rare problem f which we must still be aware when treating clavicle fractures. A careful examination identified initially subtle signs of a serious complication, which led to further investigation. This demonstrated that the clavicle was displaced under the first rib, compressing the subclavian artery as well as the BP trunks and divisions surrounding it.

Traumatic BP injury in children is rare. Dorsi *et al* estimated a prevalence of 0.1% of the 115,414 injured children they reviewed on the American National Pediatric Trauma Registry.^4^ The majority of BP injuries in this population involved high energy polytrauma with a mean injury severity score of 10 (range: 0–75). Within that, 12.4% had an associated clavicle fracture although no analysis was made as to whether this had caused the BP injury. There are no separately reported cases of BP injuries in children secondary to clavicle fractures.

Even compared with those cases reported in adults, our case is unusual. First, BP injury in the context of clavicle fracture is typically a delayed phenomenon occurring days, weeks and sometimes months after the original insult.^5–8^ Where there is immediate involvement of the BP, this is frequently secondary to a high energy traction injury rather than damage by the clavicle itself. In this case, the symptoms were progressing rapidly over a few hours.

Second, of the cases reported, BP injury secondary to clavicle fracture was caused predominantly by subclavian artery pseudoaneurysm, non-union or hypertrophic callous formation.^5,9^ Compression of the BP due to acute fracture and fragment displacement has been reported on a few occasions.^6–8,10,11^ However, this involved impingement of the BP cords by the medial end of the distal fragment, which is pulled inferiorly by the weight of the arm and medially by the muscles acting on the shoulder girdle. On the other hand, damage due to inferoposterior displacement of the proximal fragment is exceptional.^10^

Third, the features usually include progressive involvement of the medial and posterior cords.^5,6,11^ The cases described present typically with weakness and paraesthesia affecting the hand with subsequent progressive involvement of the rest of the arm. Involvement of the lateral cord is unusual and is only reported in one other case.^8^

Finally, the case described involved compromise of both the BP and the subclavian artery. There are no reports of injury to both these structures in this context. However, given their proximity, it would seem unlikely that damage to either of these would not be accompanied by damage to the other, even if at a subclinical level.

Anatomically, the BP is protected from injury by a fractured clavicle by the sternocleidomastoid, which pulls the fracture fragments away from it, and the subclavius muscle and clavipectoral fascia, which intervene between it. However, the course of the BP and axillary artery as they pass between the anterior and middle scalenes before crossing the lateral border of the first rib makes it prone to injury by compression and traction. Fractures of the middle third are most prone to displacement as it lacks the stabilising ligaments of the proximal and distal thirds.

The proximity of the axillary artery and BP mean that compromise of one of these structures is likely to involve the other. Pre-operative imaging with arteriography (CT or MRI) is essential in assessing their patency. It also highlights the requirement for the involvement of vascular and PNI specialists in their care. The availability of those with appropriate specialist skills may determine the timing of intervention. In this case, the acute presentation and the local availability of a vascular surgeon meant that the most expeditious course of action was for a PNI surgeon to come from a tertiary referral centre.

Of the reported cases of BP injury secondary to clavicle fracture, where there was gradual onset of symptoms, early decompression by closed reduction or open reduction and internal fixation was associated with a good recovery.^6–8,10,11^ Where presentation was delayed or symptoms came on immediately, then a poorer long-term outcome resulted.^10^ In the case described here, injury to the BP was complicated by involvement of the subclavian artery. It was unclear at presentation whether the neurological symptoms were a result of a laceration to the BP or a compressive ischaemic process. Early decompression was therefore deemed appropriate to prevent further BP injury.

## Conclusions

This case illustrates the rare but serious risks of neurovascular injury in clavicle fractures. In particular, it demonstrates the importance of testing and documenting neurovascular status, highlighting the awareness that good collateral circulation in children may result in a palpable radial pulse despite higher occlusion, and hence a low threshold for repeat examination and arteriography. Furthermore, it demonstrates the need for appropriate specialist input in the form of PNI and vascular surgeons, the value of early decompression of acute neurovascular injuries and the requirement to warn patients of symptoms suggestive of developing neurovascular compromise.
